# Niclosamide suppresses the expansion of follicular helper T cells and alleviates disease severity in two murine models of lupus via STAT3

**DOI:** 10.1186/s12967-021-02760-2

**Published:** 2021-02-25

**Authors:** Se Gwang Jang, Jaeseon Lee, Seung-Min Hong, Young-Seok Song, Min Jun Kim, Seung-Ki Kwok, Mi-La Cho, Sung-Hwan Park

**Affiliations:** 1 The Rheumatism Research Center, Catholic Research Institute of Medical Science, College of Medicine, The Catholic University of Korea, Seoul, Republic of Korea; 2 Division of Rheumatology, Department of Internal Medicine, Seoul St. Mary’s Hospital, College of Medicine, The Catholic University of Korea, Seoul, Republic of Korea

**Keywords:** Systemic lupus erythematosus, MRL/*lpr*, R848-induced model, Niclosamide, STAT3, Follicular helper T cells

## Abstract

**Background:**

Autoantibody production against endogenous cellular components is pathogenic feature of systemic lupus erythematosus (SLE). Follicular helper T (T_FH_) cells aid in B cell differentiation into autoantibody-producing plasma cells (PCs). The IL-6 and IL-21 cytokine-mediated STAT3 signaling are crucial for the differentiation to T_FH_ cells. Niclosamide is an anti-helminthic drug used to treat parasitic infections but also exhibits a therapeutic effect on autoimmune diseases due to its potential immune regulatory effects. In this study, we examined whether niclosamide treatment could relieve lupus-like autoimmunity by modulating the differentiation of T_FH_ cells in two murine models of lupus.

**Methods:**

10-week-old MRL/*lpr* mice were orally administered with 100 mg/kg of niclosamide or with 0.5% methylcellulose (MC, vehicle) daily for 7 weeks. TLR7 agonist, resiquimod was topically applied to an ear of 8-week-old C57BL/6 mice 3 times a week for 5 weeks. And they were orally administered with 100 mg/kg of niclosamide or with 0.5% MC daily for 5 weeks. Every mouse was analyzed for lupus nephritis, proteinuria, autoantibodies, immune complex, immune cell subsets at the time of the euthanization.

**Results:**

Niclosamide treatment greatly improved proteinuria, anti-dsDNA antibody levels, immunoglobulin subclass titers, histology of lupus nephritis, and C3 deposition in MRL/*lpr* and R848-induced mice. In addition, niclosamide inhibited the proportion of T_FH_ cells and PCs in the spleens of these animals, and effectively suppressed differentiation of T_FH_-like cells and expression of associated genes in vitro.

**Conclusions:**

Niclosamide exerted therapeutic effects on murine lupus models by suppressing T_FH_ cells and plasma cells through STAT3 inhibition.

**Supplementary Information:**

The online version contains supplementary material available at 10.1186/s12967-021-02760-2.

## Background

Systemic lupus erythematosus (SLE) is a chronic systemic autoimmune disease, with manifestations in multiple organ systems [1]. Damage to tissues in SLE results from the production of autoantibodies and immune complexes, mediated by the interplay between the innate and adaptive immune responses of autoreactive B and T lymphocytes [2, 3]. Although the exact pathogenesis of SLE has not yet been elucidated, various factors are known to be involved in driving SLE [1, 4].

Follicular helper T (T_FH_) cells are a specialized subset of CD4^+^ T cells required for germinal center (GC) formation and ultimately the development of memory B cells and high-affinity long-lived plasma cells (PCs) [5]. T_FH_ cells express high levels of the chemokine receptor CXCR5, which promotes the migration of T_FH_ cells to the B cell follicles in response to the specific ligand CXCL13 [6]. The transcription factor B cell lymphoma 6 (Bcl-6) is a master regulator of T_FH_ cells and is critical for T_FH_ cell differentiation [7]. However, additional transcription factors, including interferon-regulatory factor 4 (IRF4), basic leucine zipper transcriptional factor ATF-like (BATF), and Maf bZIP transcription factor (MAF), also play an important role in T_FH_ cell differentiation [8]. B lymphocyte-induced maturation protein 1 (Blimp-1) has been found to be the most downregulated transcription factor in T_FH_ cells, and acts as a suppressor of T_FH_ cell differentiation [9, 10]. Circulating T_FH_ cells are significantly increased in the blood of SLE patients and correlate with disease severity. In addition, T_FH_ cells are expanded in lupus-prone mice and lead to an increase in IL-21 production [4, 11].

Signal transducer and activator of transcription 3 (STAT3) plays a key role in regulating inflammation and innate/adaptive immune responses [12]. STAT3 promotes T_FH_ cell differentiation by positively regulating the transcription factors Bcl-6 and T cell factor 1 (TCF-1) [13, 14]. STAT3 signaling is enhanced by IL-6 and IL-21, which promotes the expression of Bcl-6. Previous studies have demonstrated that STAT3 is involved in the pathogenesis of SLE; indeed, a STAT3 genetic deficiency, STAT3 inhibitors, and agents that inhibit expression of STAT3 all provide a protective effect against SLE [15–18].

Several classes of agents, such as non-steroidal anti-inflammatory drugs (NSAIDs), immunosuppressants, and corticosteroids are used to treat lupus. However, these agents have a wide range of serious side effects, so their use is limited [1]. Accordingly, now, many studies are ongoing to reduce the failure of therapy by developing new agents having maximum efficacy with minimum side effects. Niclosamide is an FDA-approved anti-helminthic drug and has wide safety and few side effects, so it can be safely used for long-term period in the treatment of chronic diseases [19]. Furthermore, it is multifunctional and has also been reported by several groups to play a potential role in the treatment of various solid tumors [20, 21]. In addition, it may also be effective against autoimmune diseases and renal fibrosis due to its potential anti-inflammatory effects [22, 23]. Niclosamide is a potent inhibitor of STAT3 signaling [24]. However, the effect of niclosamide on T_FH_ cells in lupus-prone mice has not yet been investigated.

In this study, we examined whether in vivo treatment with niclosamide had a therapeutic effect in lupus-prone MRL/*lpr* and R848-induced mice by examining proportion of T_FH_ cells and PCs in the spleen and peripheral blood (PB). We additionally investigated the impact of niclosamide on T_FH_-like cells in vitro. The T_FH_ cells were sensitive to inhibition of STAT3 signaling both in vitro and in vivo. STAT3 signaling inhibition resulted in decreased numbers of T_FH_ cells in the spleen, which was correlated with an improvement in the lupus manifestation. Our findings demonstrate that niclosamide is a critical regulator of T_FH_ cells, which are a therapeutic target for SLE.

## Materials and methods

### Animals

MRL/*lpr* mice were purchased from SLC Inc. (Japan). C57BL/6 mice were purchased from OrientBio (Korea). Niclosamide (Sigma-Aldrich, St Louis, MO, USA) was resuspended in 0.5% methyl cellulose (Sigma-Aldrich, St Louis, MO, USA) for in vivo studies or in 5% DMSO for in vitro use. Female 10-week-old MRL/*lpr* mice received daily administration of vehicle (n = 7) or niclosamide (n = 7; 100 mg/kg) for 7 weeks by oral gavage. All mice were sacrificed at 16 weeks of age. Female 8-week-old C57BL/6 mice were treated via epicutaneous application of 50 μg of the TLR7 agonist resiquimod (R848; Sigma-Aldrich) dissolved in 10 μl of acetone, with or without 100 mg/kg of niclosamide daily for 4 weeks, or acetone alone as a control, to the right ear three times a week until euthanasia. All procedure of animal research were provided in accordance with the Laboratory Animals Welfare Act, the Guide for the Care and Use of Laboratory Animals and the Guidelines and Policies for Rodent experiment provided by the IACUC(Institutional Animal Care and Use Committee) in school of medicine, The Catholic University of Korea. (Approval numbers: CUMS-2018–0341-02 and 2018-0236-02).

### Enzyme-linked immunosorbent assay (ELISA)

Cytokines in sera or spleen lysates were assayed using mouse IL-6 and IL-21 Duoset ELISA kits (R&D systems, Minneapolis, MN, USA) according to the manufacturer’s instructions. The serum levels of anti-double-stranded DNA (dsDNA) IgG antibodies were measured by ELISA following the manufacturer’s instructions (Alpha Diagnostics, San Antonio, TX, USA). Total IgG, IgG1, IgG2a, and IgM levels in the sera of the mice were measured by ELISA following the manufacturer’s instructions (Bethyl Laboratories, Montgomery, TX, USA).

### Measurement of urine albumin to creatinine ratio

Urine albumin and creatinine concentrations were measured using a mouse albumin ELISA assay (Bethyl Laboratories) and a creatinine assay (R&D systems), respectively, according to the manufacturer’s directions. Urine albumin excretion was expressed as the ratio of urine albumin to creatinine (ACR).

### Histological assessment of the kidney

Kidney tissues were fixed with formalin and embedded in paraffin, cut into 3 μm sections, and stained with periodic acid–Schiff (PAS) stain. Kidney histological pathology was evaluated using the lupus nephritis classification system, as described [25].

### Immunofluorescence

Kidney tissues were stained with anti-C3 (Abcam, Cambridge, UK) at 4 °C overnight, followed by 2 h incubation with secondary antibodies conjugated to Alexa488. Nuclei were stained with 4′,6-diamidino-2-phenylindole (DAPI; Invitrogen, Carlsbad, CA, USA). Isotype control staining was conducted via probing with rat/rabbit/mouse IgG, rather than primary antibodies. Confocal images were acquired using an LSM 800 confocal microscope (Zeiss, Oberkochen, Germany).

### Flow cytometry

Spleens were minced in RPMI 1640 medium and filtered through a 40-μm cell strainer to prepare single-cell suspensions. For intracellular staining, cells were stimulated with 25 ng/mL phorbol 12-myristate 13-acetate (PMA, Sigma-Aldrich) and 250 ng/mL ionomycin (Sigma-Aldrich) with monensin-containing GolgiStop (BD biosciences, San Jose, CA, USA) for 5 h. Cells were harvested and stained with surface eFluor780-fixable viability dye (FVD) (eBioscience, Carlsbad, CA, USA), Pacific Blue-anti-CD90.2 (Biolegend, San Diego, CA, USA), PerCP-Cy5.5-anti-CD4 (Biolegend), FITC-anti-CXCR5 (Biolegend), Brilliant Violet 605-anti-PD-1 (Biolegend), APC-anti-CD19 (Biolegend), PE-anti-CD138 (BD Biosciences), and Alexa Fluor A488-anti-GL7 (eBioscience) antibodies. Blood samples were collected from the retro-orbital sinus. Red blood cell were lysed with an ammonium-chloride-potassium lysis buffer, then stained with surface eFluor 780-Fixable viability dye (eBioscience), Pacific Blue-anti-CD90.2, PerCP-Cy5.5-anti-CD4, and PE-anti-CD8 antibodies. Flow cytometric analysis was performed on a LSRII Fortessa (BD biosciences), and the data were analyzed using FlowJo software (Tree Star, Ashland, OR, USA).

### Western blot

Total protein was extracted using RIPA buffer containing Halt protease/phosphatase inhibitor cocktail (Thermo Fisher Scientific, Rockford, IL, USA). For immunoblotting, 30 μg of protein was separated using 10% sodium dodecyl sulfate polyacrylamide gel electrophoresis (SDS-PAGE), then transferred onto polyvinylidene fluoride membrane (Bio-Rad, Hercules, CA, USA), and probed with the following antibodies: anti-p-STAT3_Y705_, anti-STAT3, anti-Bcl-6, anti-TCF-1 (Cell signaling technology, Danvers, MA, USA), and anti-β-actin (Sigma-Aldrich). Subsequently, the membranes were incubated with horseradish peroxidase-conjugated goat anti-rabbit IgG or goat anti-mouse IgG (Thermo Fisher Scientific). Reactive proteins on the membrane were visualized using SuperSignal® West Pico Chemiluminescent substrate (Thermo Fisher Scientific), and the membrane was then exposed on an Amersham Imager 600 (GE healthcare, Healthcare, Chicago, IL, USA).

### Real-time PCR

Total RNA was collected using an RNA iso plus reagent (Takara, Kusatsu, Japan). Up to 1 ~ 2 µg of total RNA was converted to complementary DNA using a Transcriptor First-Strand cDNA Synthesis kit (Roche Diagnostics, Penzberg, Germany). A LightCycler 96 instrument (Roche) was used for PCR amplification and analysis. All reactions were performed with SYBR Green I Master Mix, according to the manufacturer’s instructions. Primers were designed using the web tool from GenScript® (http://www.genscript.com). Sequences are as follows (forward and reverse, respectively): *beta actin*, 5′-GGACTTCGAGCAAGAGATGG-3′ and 5′-TGTGTTGGGGTACAGGTCTTT-3′; *Bcl-6*, 5′-GCCGGCTCAATAATCTCGTGAACA-3′ and 5′-CCAGCAGTATGGAGGCACATCT-3′; *CXCR5*, 5′-ACTCCTTACCACAGTGCACC-3′ and 5′-GGAAACGGGAGGTGAACCA-3′; and *Blimp-1*, 5′-ATGGAGGACGCTGATATGAC-3′ and 5′-CCTTACTTACCACGCCAATAAC-3′. All mRNA expression levels were normalized to *beta actin* expression. Relative fold induction was calculated, following the equation 2^−(∆Cq)^ or 2^−(∆∆Cq)^, where ∆∆Cq is ∆Cq_(target)_—∆Cq_(*beta actin*)_, ∆Cq is Cq_(stimulated)_-Cq_(unstimulated)_, and Cq is the cycle at which the threshold is crossed. PCR product quality was monitored using post-PCR melting curve analysis.

### T_FH_-like cell differentiation

CD4^+^ T cells were purified from spleens of MRL/*lpr* and C57BL/6 mice using the CD4^+^ T cell Isolation Kit (Miltenyi Biotec, Bisley, UK) according to the manufacturer’s instructions. For T_FH_-like cell differentiation, purified CD4^+^ T cells were seeded at 1 × 10^6^ cells/well and were activated with mouse T-activator CD3/CD28 Dynabeads™ (Invitrogen), and treated with 20 ng/ml IL-6, 20 ng/ml IL-21, 10 μg/ml anti-IL-4, 10 μg/ml anti-IFN-γ, and 20 μg/ml anti-TGF-β (R&D Systems) for 4 days with or without niclosamide.

### Co-culture of mouse B cells and T_FH_-like cells

T_FH_-like cells from spleens of C57BL/6 mice were first cultured for 4 days with or without niclosamide. B cells were purified from spleens of C57BL/6 mice using a B cell Isolation kit (Miltenyi Biotec) according to the manufacturer’s instructions. CD19^+^ B cells were co-cultured with T_FH_-like cells (1 × 10^6^ cells/well, 1:1 ratio) and were stimulated with 50 ng/ml IL-4 (PeproTech, Rocky Hill, NJ, USA), 5 μg/ ml anti-IgM (Jackson ImmunoResearch, West Grove, PA, USA), and 5 μg/ml anti-CD40 (eBioscience) in Roswell Park Memorial Institute (RPMI)-1640 medium (Gibco, Carlsbad, CA, USA) with 10% FBS for 3 days. IgG were measured using an ELISA kit (Bethyl Laboratories).

### Statistics analysis

Statistical analyses were performed in GraphPad Prism version 7.0 software (GraphPad, San Diego, CA, USA). Statistical significance was determined by t-tests for two groups, and by one-way ANOVA with Tukey’s multiple comparisons tests for three or more groups. P < 0.05 was considered statistically significant.

## Results

### Effects of niclosamide on lupus nephritis in MRL/lpr mice

**Fig. 1:**
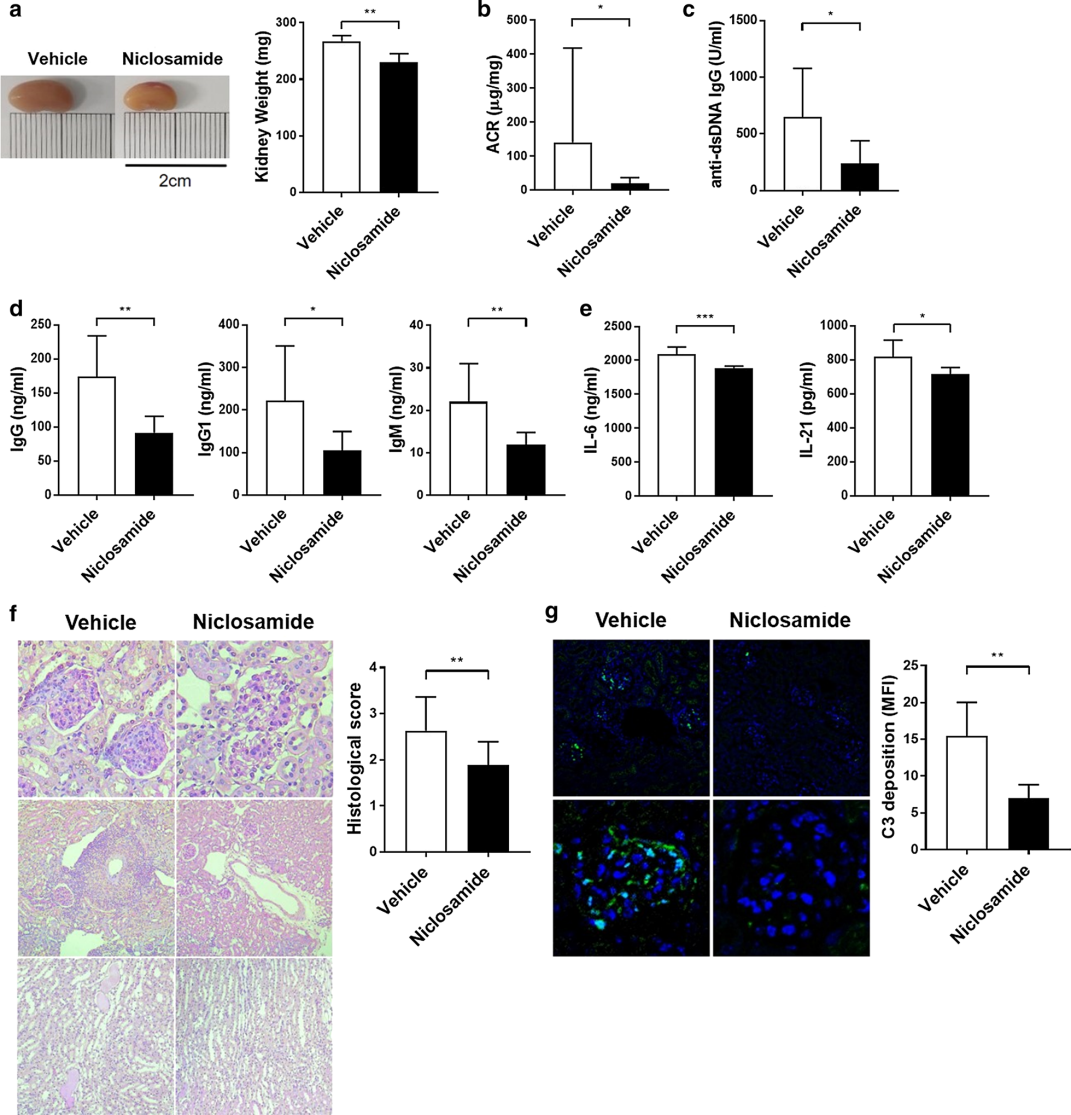
Niclosamide ameliorates disease aggravation in MRL/*lpr* mice. Female 10-week-old MRL/*lpr* mice were orally administered 0.5% methyl cellulose (vehicle, n = 7), or 100 mg/kg niclosamide (Niclosamide, n = 7) daily until they were 16-week-old. **a** Left, Representative photographs documenting the enlargement of kidneys. Right, kidney weights in vehicle, niclosamide groups. **b** Urine albumin levels normalized to creatinine. **c** Serum levels of anti-dsDNA IgG antibody. **d** Serum levels of antibody subclasses (IgG, IgG1, and IgM). **e** Serum levels of IL-6 and IL-21. **f** Left, representative photomicrographs of PAS-stained sections of kidney. Original magnification ×200 (upper), ×100 (middle, bottom). Right, histological score. **g** Left, representative immunofluorescent images of kidney C3 staining. Original magnification ×100 (upper), ×400 (bottom). Right, MFI of C3 deposition. Data shown as mean ± SD. t-test was performed. *P < 0.05, **P < 0.01, ***P < 0.001

To assess whether niclosamide ameliorates clinical features of LN, we orally administered 10-week-old MRL/*lpr* mice with niclosamide or vehicle daily for 7 weeks. While the kidney weights increased with disease progression in this spontaneous lupus model, niclosamide treatment inhibited kidney enlargement (Fig. 1a). Proteinuria, one of the major measures of LN, was reduced upon niclosamide treatment, in contrast to the vehicle (Fig. 1b). We next measured autoantibodies in these mice, which is the hallmark of SLE. Niclosamide treatment decreased serum levels of anti-dsDNA IgG (Fig. 1c), in addition to the serum levels of IgG, IgG1, and IgM (Fig. 1d). IL-6 and IL-21 are pro-inflammatory cytokine for lupus progression, and also important regulators of T_FH_ cell generation. We found that niclosamide treatment significantly decreased the serum levels of IL-6 and IL-21 (Fig. 1e).

Furthermore, MRL/*lpr* mice developed severe renal injury, which was characterized by diffuse glomerular, perivascular, and interstitial mononuclear cell infiltration, tubular cast deposition, increased mesangial matrix, and immune complex deposition. Niclosamide ameliorated these renal pathological features, as judged by changes in the histological scores and C3 immune complex deposition (Fig. 1f, g). These data suggest that niclosamide treatment significantly prevented LN in MRL/*lpr* mice.

### Niclosamide inhibits CD90.2^+^ T cell, DNT cell, T_FH_ cell and PC differentiation in MRL/lpr mice

**Fig. 2:**
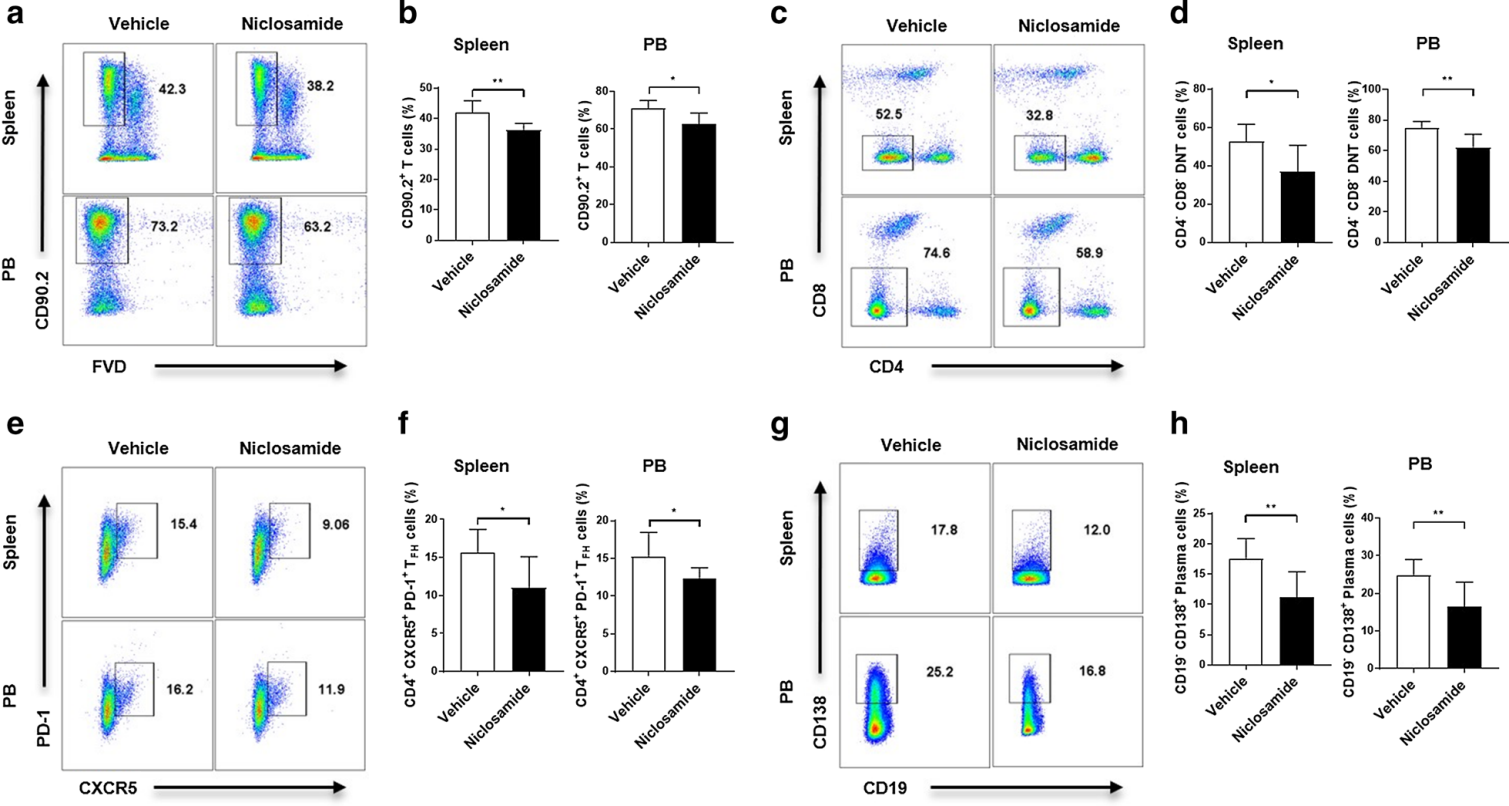
Niclosamide modulates the proportion of CD90.2^+^ T cells, DNT cells, T_FH_ cells and PCs in the spleen and PB of MRL/*lpr* mice. Spleen and PB samples were collected from 16-week-old mice. **a** comparison of the FVD^−^ and CD90.2^+^ T-cell proportion. The gating strategies were: lymphocytes as FSC-A vs SSC-A, live cells as FVD-A vs CD90.2-A **b** Bar graphs show mean ± SD. **c** Altered proportion of DNT cells (CD4^−^ CD8^−^ gated on FVD^−^ CD90.2^+^) by niclosamide. **d** Bar graphs show mean ± SD. **e** Data show proportion of T_FH_ cells (CXCR5^+^ PD-1^+^ gated on CD4 +). **f** Bar graphs show mean ± SD. **g** Data show proportion of PCs (CD19^−^ CD138^+^ gated on CD19^−^). **h** Bar graphs show mean ± SD. t-test was performed. *P < 0.05, **P < 0.01

To investigate the cell types related to the decreased disease severity by niclosamide in MRL/*lpr* mice, we analyzed the proportion of T cell and B cell subsets in spleens and PB. CD90.2^+^CD4^−^CD8^−^ double-negative T cells (DNT cells) are expanded in SLE patients, as well as massive splenomegaly and lymphadenopathy caused by hyperproliferation of DNT cells in MRL/*lpr* mice [26]. T_FH_ cells, defined as CD4^+^ CXCR5^+^ PD-1^+^, play a crucial role in GC formation and the production of antigen-specific memory B cells and PCs, thereby contributing to SLE disease progression [4, 27]. We analyzed the proportion of CD90.2^+^ T cells, DNT cells, T_FH_ cells and PCs in the spleens and PB of MRL/*lpr* mice. Niclosamide treatment had a significantly lower proportion of spleen CD90.2^+^ T cells than the vehicle treatments. The proportion of PB CD90.2^+^ T cells was significantly decreased in the niclosamide treatment compared with the vehicle treatment (Fig. 2a, b). Likewise, the spleen DNT cells proportion was decreased by niclosamide treatment (Fig. 2c, d). We found that the proportion of T_FH_ cells in both samples from these mice were significantly decreased by niclosamide treatment (Fig. 2e, f). In addition, The proportion of Th1, Th17, and regulatory T (Treg) cells were analyzed in spleen samples using flow cytometry. The alteration of the spleen Th1 and Th17 cells proportion was not significant (Additional file 1: Figure S1a-c). However, Treg cells, characterized by immunomodulatory capabilities, were significantly increased by niclosamide treatment (Additional file 1: Figure S1d and e). The Treg/Th17 ratio was significantly increased in the niclosamide group compared with the vehicle group in spleen (Additional file 1: Figure S1f). In addition to, the proportion of PCs in both samples from these mice were significantly reduced by niclosamide treatment (Fig. 2g, h). These data indicate that niclosamide could suppress the number of CD90.2^+^ cells, DNT cells, T_FH_ cells and PCs in the spleens and PB of MRL/*lpr* mice.

### Niclosamide regulates major transcription factors involved in T_FH_ cell differentiation

**Fig. 3:**
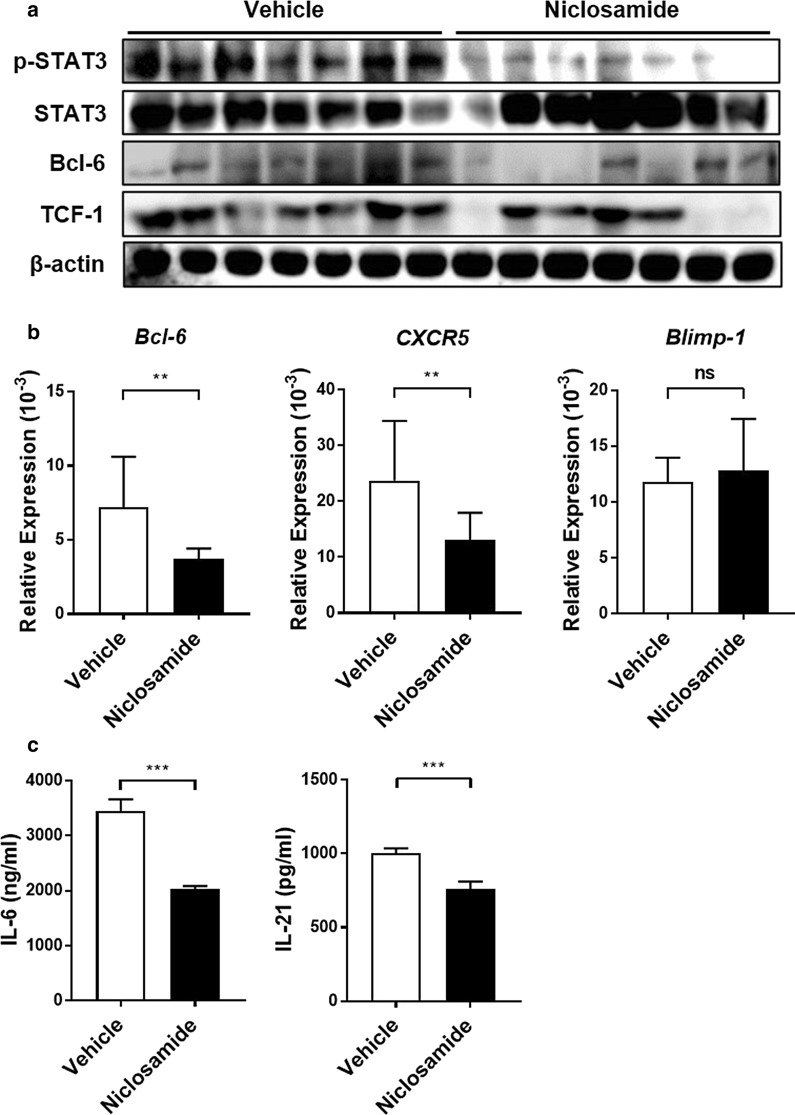
Niclosamide treatment reduces T_FH_-associated gene and cytokine levels in the spleens of MRL/*lpr* mice. **a** p-STAT3, Bcl-6, and TCF-1 expression in the spleen were analyzed by western blot. **b** mRNA expression levels of *Bcl-6*, *CXCR5*, and *Blimp-1* in CD4^+^ T cells purified from spleens for each group of mice were measured by real-time PCR. **c** The levels of IL-6 and IL-21 in the spleen homogenates were detected by ELISA. Data shown as mean ± SD. t-test was performed. **P < 0.01, ***P < 0.001, ns: not significant

We next analyzed the activity of the STAT3/Bcl-6/TCF-1 pathways in the spleens of each animal group to elucidate the molecular mechanisms regulating the effects of niclosamide treatment. Western blot analysis revealed that expression of p-STAT3/Bcl-6/TCF-1 was significantly decreased in the splenocytes of niclosamide-treated mice as compared to vehicle-treated mice (Fig. 3a). Previous studies have shown that Bcl-6, CXCR5, and Blimp-1 are important regulators of T_FH_ cell differentiation [9, 28]. Niclosamide significantly reduced *Bcl-6* and *CXCR5* mRNA expression, while the mRNA levels of *Blimp-1*, which acts as a T_FH_ cell differentiation suppressor, was not significantly increased in spleen CD4^+^ T cells (Fig. 3b). In addition, niclosamide treatment significantly decreased the spleen levels of IL-6 and IL-21 (Fig. 3c). Collectively, these results indicate that niclosamide is effective in regulating T_FH_ cell-related factors.

### STAT3 is important for T_FH_-like cell differentiation and B cell IgG production in vitro

**Fig. 4:**
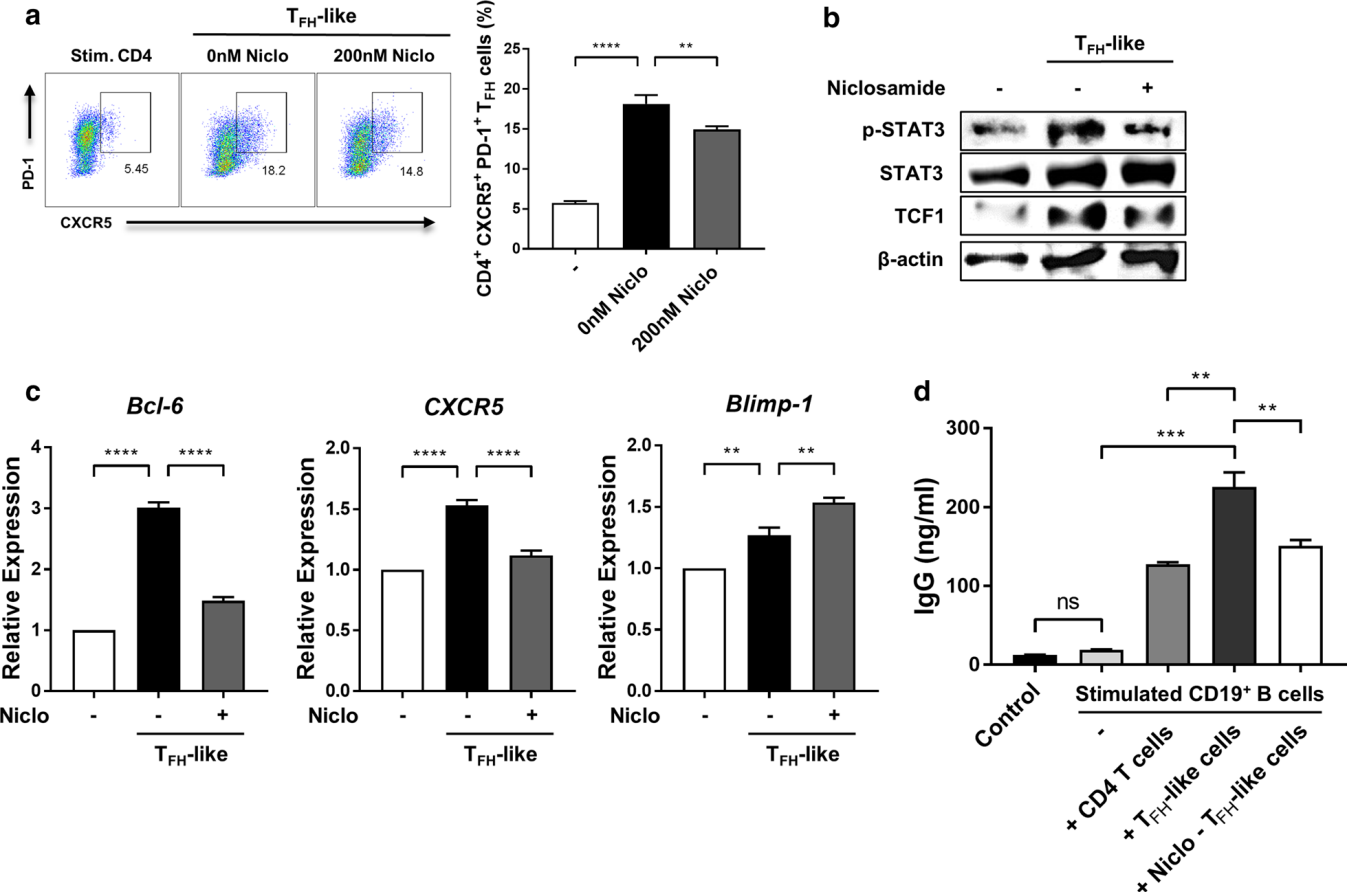
Niclosamide inhibits T_FH_-like cell differentiation and B cell IgG production in vitro. Naive CD4^+^ T cells were purified from the spleens of MRL/*lpr* and C57BL/6 mice. For T_FH_-like cell differentiation, purified CD4^+^ T cells were activated with mouse T-activator CD3/CD28 Dynabeads, and treated with 20 ng/ml IL-6, 20 ng/ml IL-21, 10 μg/ml anti-IL-4, 10 μg/ml anti-IFN-γ, and 20 μg/ml anti-TGF-β for 4 days with or without niclosamide. **a** Left, T_FH_-like cells (CXCR5^+^PD-1^+^, gated on CD4^+^) isolated from MRL/*lpr* mice were analyzed by flow cytometry. Right, the percentage of T_FH_-like cells is shown. **b** p-STAT3 and TCF-1 expressions in T_FH_-like cells isolated from MRL/*lpr* mice were analyzed by western blot. **c** mRNA expression levels of *Bcl-6*, *CXCR5*, and *Blimp-1* in T_FH_-like cells isolated from MRL/*lpr* mice were measured by real-time PCR. **d** T_FH_-like cells and B cells isolated from C57BL/6 mice were co-cultured with or without T_FH_-like cells for 3 days, and then the concentrations of IgG in the supernatants were detected by ELISA. Data shown as mean ± SD. One-way ANOVA was performed. **P < 0.01, ***P < 0.001, ****P < 0.0001, ns: not significant

We further investigated the influence of niclosamide on T_FH_-like cell differentiation in vitro, and found that niclosamide could inhibit T_FH_-like cell differentiation (Fig. 4a). Niclosamide treatment reduced the protein levels of p-STAT3 and TCF-1 in cultured T_FH_-like cells (Fig. 4b). In addition, niclosamide inhibited mRNA expression of *Bcl-6* and *CXCR5*, and increased *Blimp-1* during the differentiation of T_FH_-like cells (Fig. 4c). We next examined whether T_FH_-like cells might play a pathogenic role in SLE by supporting the production of autoantibodies. When B cells were cultured with T_FH_-like cells, IgG production was significantly increased compared to CD4^+^ T cells. However, when B cells were cultured with niclosamide-treated T_FH_-like cells, IgG production markedly decreased (Fig. 4d). By enhancing antibody production in vitro, T_FH_-like cells thus demonstrated their capacity to promote an antibody response. Overall, these results indicate that niclosamide not only inhibits T_FH_-like cell differentiation, but also B cell IgG production.

### Effects of niclosamide on lupus nephritis in an R848-induced mouse model

**Fig. 5:**
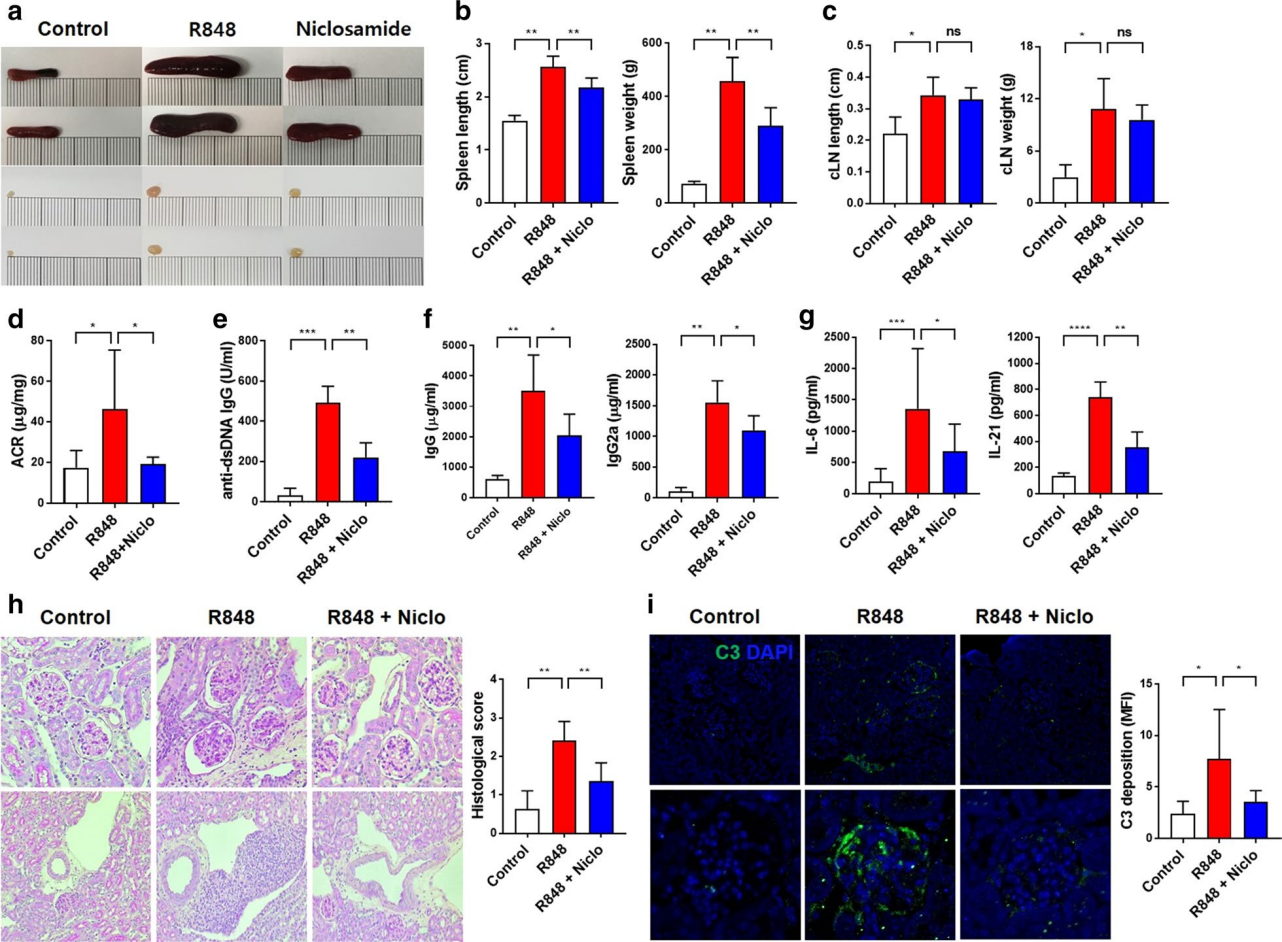
Niclosamide ameliorates disease aggravation in R848-induced mice. Female 8-week-old C57BL/6 mice were treated with 50 μg of the TLR7 agonist R848 or control (acetone) three times weekly and were orally administered 0.5% methyl cellulose (control, R848, n = 4–6), or 100 mg/kg niclosamide (niclosamide, n = 7) daily until they were 12-week-old. **a** Representative photographs documenting the enlargement of spleens and cLNs. **b** Spleen lengths and weights in each group. **c** cLN lengths and weights in each group. **d** Urine albumin levels normalized to creatinine. **e** Serum levels of anti-dsDNA IgG antibody. **f** Serum levels of antibody subclasses (IgG, IgG2a). **g** Serum levels of IL-6, IL-21. **h** Left, representative photomicrographs of PAS-stained sections of kidney. Original magnification ×200 (upper), ×100 (bottom). Right, histological scores. **i** Left, representative immunofluorescent images of kidney C3 staining. Original magnification ×100 (upper), ×400 (bottom). Right, MFI of C3 deposition. Data shown as mean ± SD. One-way ANOVA was performed. *P < 0.05, **P < 0.01, ***P < 0.001, ****P < 0.0001, ns: not significant

To further verify our findings in a different murine model, wild-type C57BL/6 mice received topical treatment on their right ears with the TLR-7 agonist R848 3 times weekly for 5 weeks. We orally administered 8-week-old R848-induced mice with niclosamide or vehicle daily for 5 weeks. R848-induced mice exhibited splenomegaly, while niclosamide treatment significantly reduced the spleen enlargement in these animals, although the cervical lymph node (cLN) size was not significantly different (Fig. 5a–c). The R848 group developed proteinuria compared with the control group, but the niclosamide-treated animals showed significantly diminished proteinuria compared with the R848 group (Fig. 5d). Serum levels of anti-dsDNA IgG, IgG, and IgG2a were significantly increased in the R848-induced mice as compared to the control mice. However, niclosamide treatment significantly decreased the levels of these factors (Fig. 5e, f). In addition, serum levels of IL-6 and IL-21 were increased following R848 stimulation, while niclosamide treatment prevented these increases (Fig. 5g). R848-induced mice also developed enlarged hypercellular glomeruli, an increase in the mesangial matrix, moderate perivascular mononuclear cell infiltration, glomerular basement membrane thickening, and immune complex deposition. Niclosamide relieved these renal pathological features, decreased histological scores, and inhibited C3 immune complex deposition (Fig. 5h, i). These results suggest that mice topically treated with R848 developed systemic autoimmunity, but this effect was significantly alleviated by niclosamide treatment.

### Niclosamide inhibits CD4^+^ T cell, T_FH_ cell, GC B cell, and PC differentiation in R848-induced mice

**Fig. 6:**
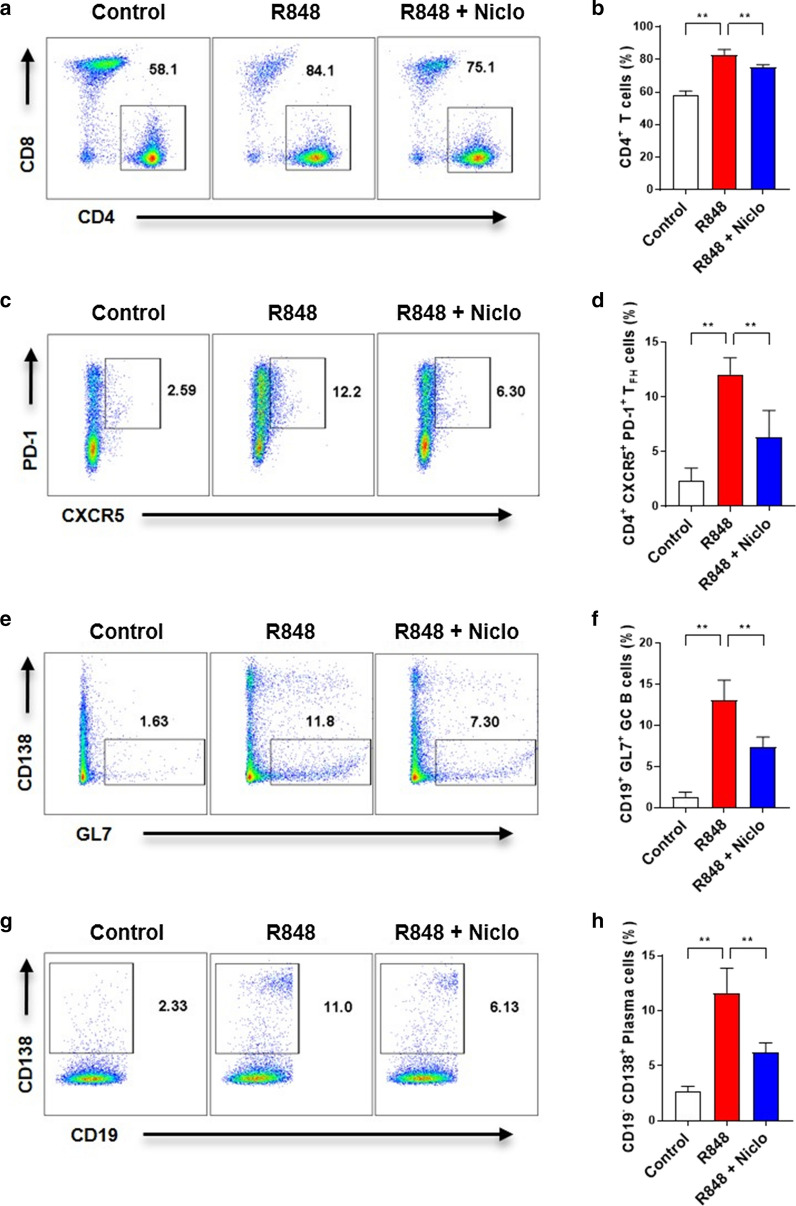
Niclosamide reduces the proportion of CD4^+^ T cells, T_FH_ cells, GCs, and PCs in spleens of R848-induced mice. Spleen samples were collected from 12-week-old mice. **a** Data show proportion of CD4^+^ T cells (CD4^+^ CD8^−^ gated on FVD^−^ CD90.2^+^). **b** Bar graphs show mean ± SD. **c** Data show proportion of T_FH_ cells (CXCR5^+^ PD-1^+^ gated on CD4^+^). **d** Bar graphs show mean ± SD. **e** Data show proportion of GC B cells (CD138^−^ GL7^+^ gated on CD19^+^). **f** Bar graphs show mean ± SD. **g** Data show proportion of PCs (CD19^−^ CD138^+^ gated on CD19^−^). **h** Bar graphs show mean ± SD. One-way ANOVA was performed. **P < 0.01

We next sought to determine whether the proportion of T cell and B cell subsets expanded in response to R848 treatment, and whether this response would be inhibited by niclosamide. CD4^+^ T cells, T_FH_ cells, GC B cells, and PCs were significantly increased in the spleens of R848-induced mice (Fig. 6). However, niclosamide significantly decreased the proportion of CD4^+^ T cells (Fig. 6a, b). The proportion of T_FH_ cells was also significantly decreased by niclosamide treatment (Fig. 6c, d). Additionally, when the T cell subsets analysis was performed, Th1 cells were significantly decreased by niclosamide, while Th17 cells were not affected (Additional file 2: Figure S2). GC B cell proportion in R848-induced mice were inhibited by niclosamide (Fig. 6e, f). The proportion of PCs in these mice was significantly reduced by niclosamide treatment (Fig. 6g, h). These data suggest that R848 enhanced CD4^+^ T cell, T_FH_ cell, GC B cell, and PC differentiation, but the expansion of these cells was dramatically prevented by niclosamide.

## Discussion

Autoreactive antibody production is one of the hallmarks of SLE and plays a critical pathogenic role in LN [29]. T_FH_ cells promote autoantibody-producing B cell differentiation in autoimmune diseases. Abnormal expansion of T_FH_ cells is a common feature of lupus patients with active disease and is also found in spontaneous and induced models of murine lupus, which suggests that T_FH_ cells affect lupus pathogenesis [30, 31]. We hypothesized that STAT3-mediated regulation of T_FH_ cells could potentially limit the pathogenicity of the disease. To address this, we used MRL/*lpr* mice and R848-induced mice. MRL/*lpr* mice spontaneously develop a severe lupus-like disease that shares several of the immunologic and clinical manifestations of human lupus [32]. Several studies have shown that T_FH_ cells are expanded in the spleens of MRL/*lpr* mice [33, 34]. R848 is an immune-response modifier that activates immune cells through Toll-like receptors (TLRs) 7 and 8 [35]. Epicutaneous application of R848 to C57BL/6 mice induces the development of lupus-like phenotypes, including mild LN [36]. Activation of plasmacytoid dendritic cells (pDCs) by R848 play a critical role in antigen presentation and T cell responses [37]. pDCs induce T_FH_ cell differentiation through cytokine secretion, such as type I IFN and IL-6 [27, 38]. Although the T_FH_ cell differentiation mechanisms differ between the two mouse models, the differentiation of these cells was significantly inhibited by niclosamide, thereby alleviating the symptoms of lupus.

STAT3 serves multiple biological functions, including playing roles in proliferation, differentiation, and survival through cytokine-mediated signaling in various cells. STAT3 is especially critical for T_FH_ and Th17 cell differentiation [39, 40]. Th17 cells significantly contribute to SLE pathogenesis [41], while inhibition of STAT3 delays the development of lupus pathogenesis by suppressing Th17 cell differentiation [42]. However, we found that niclosamide did not reduce Th17 cell numbers. In addition, DNT cells, which play a crucial pathological role in the development of lupus disease, are expanded in MRL/*lpr* mice and patients with SLE. In MRL/*lpr* mice, DNT cells have been found to be the most expanded T cell subset, which lead to splenomegaly and lymphadenopathy [26]. Although we suggested that splenomegaly and lymphadenopathy were not significantly reduced by niclosamide treatment, DNT cell numbers were significantly inhibited. In further studies, we will explore the mechanisms of DNT cell regulation by niclosamide.

Blimp-1 is a well-known antagonist of Bcl-6 that can directly inhibit Bcl-6 expression in T and B cells [43]. Conversely, Bcl-6 can suppress the expression of Blimp-1 [44]. Blimp-1 is a key transcription factor for regulating PC differentiation in B cell lines [45]. Genome-wide association studies have identified multiple SLE-related genes and new susceptibility loci, including Blimp-1 [46]. As mentioned previously, Bcl-6 is strongly upregulated in T_FH_ cells [7], while conversely Blimp-1 is the most downregulated transcription factor in T_FH_ cells [47]. Bcl-6 and Blimp-1 are mutual master transcription factors regulating T_FH_ cell differentiation, and the presence of Bcl-6 coupled with the absence of Blimp-1 is required for T_FH_ cell differentiation [9]. In our study, we found that Bcl-6 was significantly decreased in CD4^+^ T cells by niclosamide administration in MRL/*lpr* mice, while Blimp-1 was not significantly increased. Further, during T_FH_-like cell differentiation in vitro, Bcl-6 was significantly decreased and Blimp-1 was significantly increased by niclosamide treatment.

TCF-1 is crucial for T cell development, as this transcription factor promotes the formation of memory CD8^+^ T cells, Th2 cells, and T_FH_ cells. TCF-1 is highly expressed and regulates the early stages process of T_FH_ cell differentiation [13]. Xu et al. reported that TCF-1 functions as an upstream regulator of the Bcl-6 / Blimp-1 axis, critical for T_FH_ differentiation [48]. Certain cytokines secreted by immune cells in the disease milieu, such as IL-6 and IL-21 [49, 50], might induce the immediate upregulation of TCF-1 expression through STAT3 signaling. We found that niclosamide decreased serum levels of IL-6 and IL-21. Niclosamide also inhibited TCF-1 expression in the spleens of MRL/*lpr* mice and in T_FH_-like cells.

Niclosamide induce cell cycle arrest, growth inhibition and apoptosis in cancer cells by targeting multiple signaling pathways such as, Wnt/β-catenin, STAT3, mTOR, Notch signaling pathways and mitochondria metabolic pathways [51]. Han et al. demonstrated that niclosamide ethanolamine alleviated lupus nephritis in MRL/*lpr* mice, and regulated mitochondrial biogenesis and energy metabolism in the kidney [52]. An important feature of mitochondria is it can regulate activation, differentiation, and survival of immune cells such as, T cell, B cell, macrophage, DC in autoimmune disease [53]. However, the authors work focused primarily on the kidney and did not extensively address potential immunological mechanisms. In our study, it is necessary to further study the mechanism by which niclosamide induced mitochondria function change affects T_FH_ cells.

## Conclusion

The present study showed that niclosamide significantly alleviated SLE-like characteristics in MRL/*lpr* and R848-induced mice models. Our data also demonstrated the regulatory function of niclosamide on T_FH_ cells in vivo and in vitro. Targeting STAT3 signaling using niclosamide could be an effective therapy for treating LN.

## Supplementary Information

**Additional file 1.** Additional figures.

## Data Availability

All data are available in the manuscript or upon request to the authors.

## Abbreviations

ACR: Albumin to creatinine ratio
BATF: Basic leucine zipper transcriptional factor ATF-like
Bcl-6: B cell lymphoma 6
Blimp-1: B lymphocyte-induced maturation protein 1
cLN: Cervical lymph node
DAPI: 4′,6-Diamidino-2-phenylindole
DNT: Double-negative T
FVD: Fixable viability dye
dsDNA: Double-stranded DNA
GC: Germinal center
IRF4: Interferon regulatory factor 4
MC: Methyl-cellulose
NSAIDs: Non-steroidal anti-inflammatory drugs
PAS: Periodic acid–Schiff
PB: Peripheral blood
PCs: Plasma cells
pDC: Plasmacytoid dendritic cells
PMA: Phorbol 12-myristate 13-acetate
SDS-PAGE: Sodium dodecyl sulfate polyacrylamide gel electrophoresis
SLE: Systemic lupus erythematosus
STAT3: Signal transducer and activator of transcription 3
TCF-1: T cell factor 1
TFH: Follicular helper T
TLR7: Toll-like receptor 7
Treg: Regulatory T
