# Supplementary material for: Niclosamide suppresses the expansion of follicular helper T cells and alleviates disease severity in two murine models of lupus via STAT3
Source: J Transl Med. 2021 Feb 25;19:86. doi: 10.1186/s12967-021-02760-2 (PMC7908700; doi:10.1186/s12967-021-02760-2)

**Additional Figures**

**Figure S1.** **Niclosamide promotes the proportion of CD25^+^Foxp3^+^CD4^+^ T cells (Treg cells) in MRL/*lpr* mice.**

Spleen samples were collected from each group of 16-week-old mice. All samples were analyzed by flow cytometry. **a** Data show the proportion of Th1 (IFNγ^+^) cells or Th17 (IL-17^+^) cells gated by CD4^+^ T cells. **b, c** Bar graphs show mean ± SD. **d** Data show proportion of CD25^+^Foxp3^+^ regulatory T cells gated by CD4^+^ T cells. **e** Bar graphs show mean ± SD. **f** The ratio of Treg/Th17 in the spleen. * = *P* < 0.05, ns: not significant.


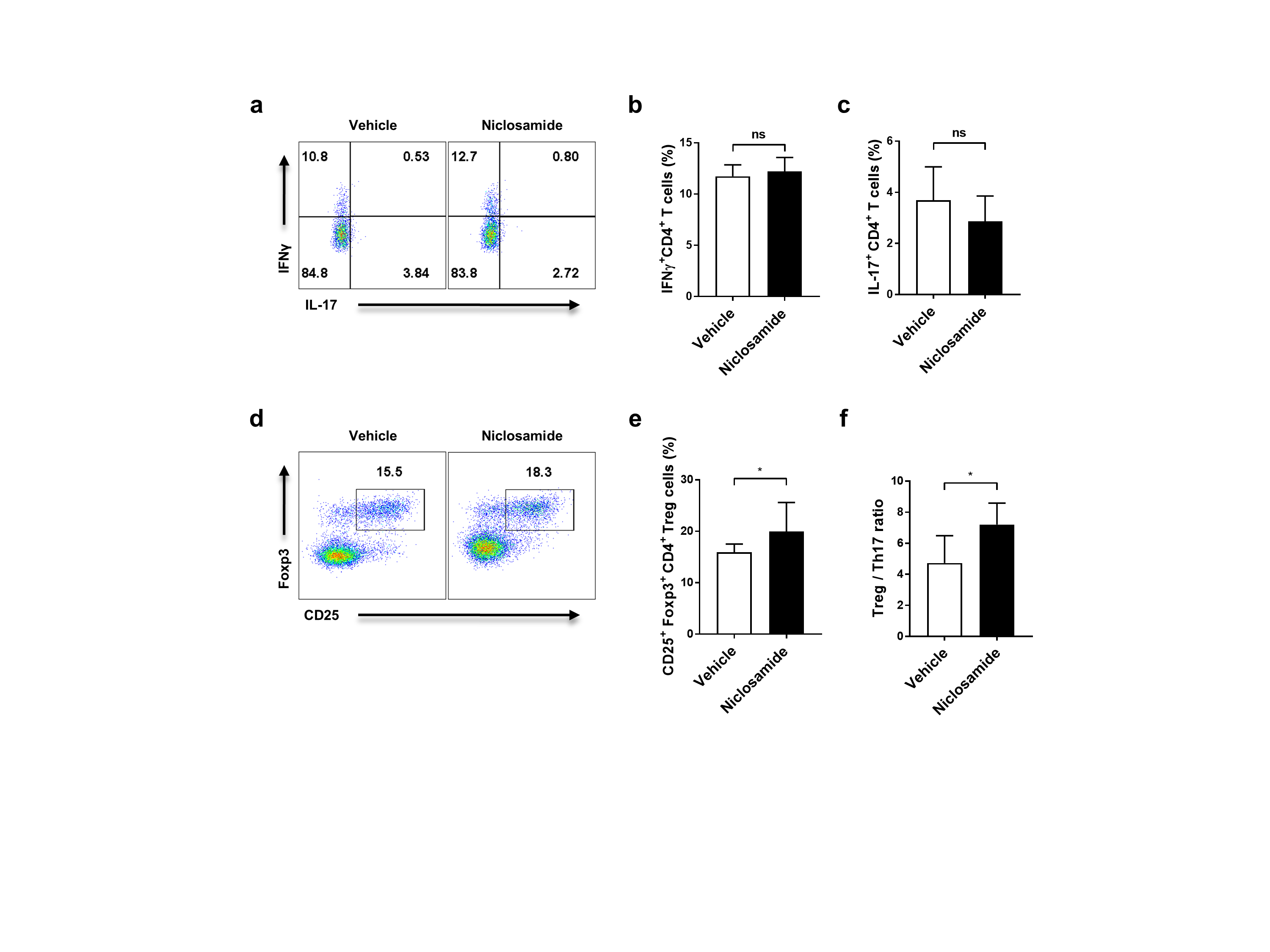


**Figure S2.** **Niclosamide inhibits the proportion of Th1 cells in R848-induced mice.**

Spleen samples were collected from each group of 12-week-old mice. All samples were analyzed by flow cytometry. **a** Data show proportion of Th1 (IFNγ^+^) cells or Th17 (IL-17^+^) cells gated by CD4^+^ T cells. **b, c** Bar graphs show mean ± SD. ns: not significant, * = *P* < 0.05, ** = *P* < 0.01, ns: not significant.


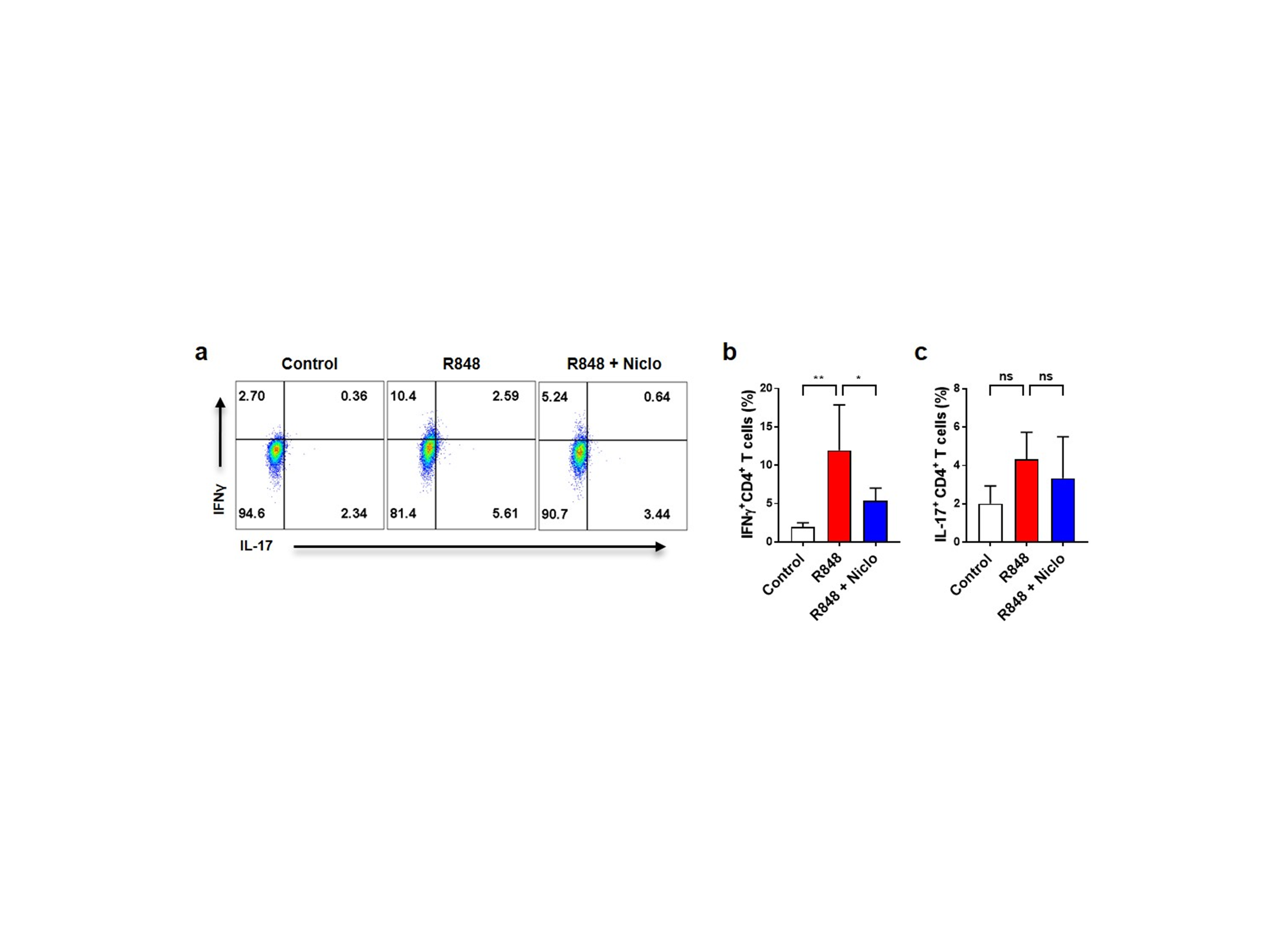

Supplement: Supplementary file 1 — Additional file 1. Additional figures. [file 12967_2021_2760_MOESM1_ESM.docx]
